# ^13^C Pyruvate Transport Across the Blood-Brain Barrier in Preclinical Hyperpolarised MRI

**DOI:** 10.1038/s41598-018-33363-5

**Published:** 2018-10-10

**Authors:** Jack J. Miller, James T. Grist, Sébastien Serres, James R. Larkin, Angus Z. Lau, Kevin Ray, Katherine R. Fisher, Esben Hansen, Rasmus Stilling Tougaard, Per Mose Nielsen, Jakob Lindhardt, Christoffer Laustsen, Ferdia A. Gallagher, Damian J. Tyler, Nicola Sibson

**Affiliations:** 1 Department of Physiology, Anatomy, and Genetics, University of Oxford, Oxford, UK; 2 Department of Physics, Clarendon Laboratory, University of Oxford, Oxford, UK; 3 Oxford Centre for Clinical Magnetic Resonance Research, John Radcliffe Hospital, Oxford, UK; 4 Department of Radiology, University of Cambridge, Cambridge, UK; 5 Cancer Research UK and Medical Research Council Oxford Institute for Radiation Oncology, Department of Oncology, University of Oxford, Oxford, UK; 6 School of Life Sciences, University of Nottingham, Nottingham, UK; 7 Physical Sciences, Sunnybrook Research Institute, Toronto, ON Canada; 8 Department of Medical Biophysics, University of Toronto, Toronto, Canada; 9 Wellcome Centre for Integrative Neuroimaging, FMRIB, Nuffield Department of Clinical Neurosciences, University of Oxford, Oxford, UK; 10 Department of Chemistry, University of Oxford, Oxford, UK; 11 MR Research Centre, Department of Clinical Medicine, Aarhus University, Aarhus, Denmark; 12 Department of Cardiology, Aarhus University Hospital, Skejby, Aarhus, Denmark

## Abstract

Hyperpolarised MRI with Dynamic Nuclear Polarisation overcomes the fundamental thermodynamic limitations of conventional magnetic resonance, and is translating to human studies with several early-phase clinical trials in progress including early reports that demonstrate the utility of the technique to observe lactate production in human brain cancer patients. Owing to the fundamental coupling of metabolism and tissue function, metabolic neuroimaging with hyperpolarised [1-^13^C]pyruvate has the potential to be revolutionary in numerous neurological disorders (e.g. brain tumour, ischemic stroke, and multiple sclerosis). Through the use of [1-^13^C]pyruvate and ethyl-[1-^13^C]pyruvate in naïve brain, a rodent model of metastasis to the brain, or porcine brain subjected to mannitol osmotic shock, we show that pyruvate transport across the blood-brain barrier of anaesthetised animals is rate-limiting. We show through use of a well-characterised rat model of brain metastasis that the appearance of hyperpolarized [1-^13^C]lactate production corresponds to the point of blood-brain barrier breakdown in the disease. With the more lipophilic ethyl-[1-^13^C]pyruvate, we observe pyruvate production endogenously throughout the entire brain and lactate production only in the region of disease. In the *in vivo* porcine brain we show that mannitol shock permeabilises the blood-brain barrier sufficiently for a dramatic 90-fold increase in pyruvate transport and conversion to lactate in the brain, which is otherwise not resolvable. This suggests that earlier reports of whole-brain metabolism in anaesthetised animals may be confounded by partial volume effects and not informative enough for translational studies. Issues relating to pyruvate transport and partial volume effects must therefore be considered in pre-clinical studies investigating neuro-metabolism in anaesthetised animals, and we additionally note that these same techniques may provide a distinct biomarker of blood-brain barrier permeability in future studies.

## Introduction

Hyperpolarisation methods overcome the fundamental Boltzmann limitation on the signal-to-noise ratio of Magnetic Resonance (MR) experiments through the exogenous creation of nuclear polarisation. The introduction of the dissolution Dynamic Nuclear Polarisation (dDNP) method for metabolic imaging *in vivo* has generated a new field of metabolic imaging, in which ^13^C labelled metabolites are hyperpolarised at cryogenic temperatures (~0.8 K), rapidly melted, and introduced into a living system, with their subsequent metabolic behaviour quantified through MR^1–3^. There are numerous distinct properties that an injectable hyperpolarised probe must possess in order to be of biomedical utility: a long nuclear *T*_1_, glassing ability, and miscibility with commonly-used electronic free radicals are required from a technical perspective, whereas minimal toxicity, rapid vascular transportation, uptake across the plasma membrane, and subsequent cellular metabolism on the timescale of *T*_1_ are required for the probe to be of subsequent biomedical utility^4–6^.

Owing to its favourable nuclear properties and its metabolic location at the end of glycolysis and entrance to the TCA cycle, hyperpolarised [1-^13^C]pyruvate is currently in the process of transitioning to the clinic as a powerful probe for the diagnosis of disease and quantification of its response to treatment. Following dissolution and injection, hyperpolarised [1-^13^C]pyruvate is transported through the body by the systemic circulation prior to its active transport into cells through proton-coupled monocarboxylate transporters (MCTs), particularly MCT-1^7^, and its subsequent cytosolic or mitochondrial metabolism, all within the lifetime of the hyperpolarised experiment. Uniquely in the brain, MCT-1 is expressed at both the luminal and abluminal membranes of the endothelial cells comprising the Blood-Brain Barrier (BBB), facilitating pyruvate transport across the blood brain barrier^8,9^.

In the heart, hyperpolarised [1-^13^C]pyruvate has been shown to be of utility in monitoring myocardial ischaemia^4,10^, in visualising the pathogenesis of heart failure^11^, and other cardiac pathologies. Owing to rapid vascular transport and a high metabolic demand, it is possible to dynamically resolve cardiac metabolism at comparatively high spatial and temporal resolution in rodents^12,13^, pigs^14–19^, and, recently, humans^20^. Likewise, hyperpolarized [1-^13^C]pyruvate has been used to probe renal metabolism in pathophysiology such as diabetes and following acute kidney injury^21–25^; and physiological hepatic metabolism in the fasted and fed states^26^, after consuming alcohol^27^, and in hepatocarcinoma^28,29^. In the heart, kidney and liver, hyperpolarised [1-^13^C]pyruvate metabolism can therefore be spatially and temporally resolved with a good signal-to-noise ratio at a physiologically relevant spatial resolution, and addresses pertinent biological and medical questions following the clear uptake and metabolism of the probe. In this work, we quantitatively assess the metabolism of [1-^13^C]pyruvate and ethyl-[1-^13^C]pyruvate in the brain in comparison to other organs, in the context of the metabolic dysregulation present in cancer.

As one of the hallmarks of cancer is a profound shift away from oxidative metabolism towards aerobic glycolysis, deemed the Warburg effect^30^, DNP with hyperpolarized [1-^13^C]pyruvate is uniquely positioned to quantify real-time exchange of the injected probe into [1-^13^C]lactate. Consequently, amongst other situations, the technique has been used extensively to study tumour metabolism in cell suspensions *in vitro*^31,32^, in xenografts^33,34^, as well as endogenous or spontaneously arising disease models such as the TRAMP mouse model of prostate cancer^35–37^. Furthermore, as the apparent lactate label exchange rate correlates with tumour aggressiveness and hence prognosis^38^, the key role of metabolism within tumour cells means that therapeutic interventions have been shown to substantially alter the apparent flux of [1-^13^C]pyruvate to [1-^13^C]lactate after radiotherapy^39,40^ or chemotherapy^38,41–43^ prior to a concomitant reduction in tumour volume as visualised by MR, even in the presence of pseudo-progression. Consequently, the initial human experiences of hyperpolarised pyruvate were performed in biopsy-proven prostate cancer patients, with the metabolic signature prospectively predicting bilateral disease in one instance^44^. Following the improved accessibility of the technique to other sites, further human studies have been undertaken and report on the feasibility of the technique for imaging lactate production in human brain^45,46^.

Metabolic alterations are additionally implicated in a large number of relevant and prevalent neurological disorders, such as increased lactate detected via ^1^H spectroscopy in multiple sclerosis lesions^47^, in experimental models of Parkinson’s disease^48^, in Alzheimer’s disease patients^49^, following traumatic brain injury^50^, in ischaemic stroke^51,52^ (correlating with the degree of neuronal loss^53^), and in leukoencephalopathy following heroin usage^54^. In stark contrast to the wide range of potential indications for the utility of hyperpolarised [1-^13^C]pyruvate imaging in neurology, there remains a small, but growing body of work where hyperpolarisation techniques have been applied to probe neurological metabolism *in vivo*. Previous work in the brain with hyperpolarized compounds has almost exclusively examined the healthy brain in animals from rodents^55,56^ to simians^57^, and predominantly one pathology: experimental models of primary brain cancer, typically glioblastoma multiforme^58^, as well as one early study in stroke^59^. In primary brain cancer, both [1-^13^C]pyruvate, [2-^13^C]pyruvate^58^ and ethyl-[1-^13^C]pyruvate have all been used to probe metabolism in naïve brain^21,60–62^ compared to contralateral large diffuse glioma^63^, and the hyperpolarized lactate flux has been shown to be substantially reduced following radiotherapy^64^, useful in reporting on the IDH-1 status of the disease^65^. Likewise, inspired by thermal equilibrium studies, hyperpolarised ^13^C -acetate has been infused into the rodent brain and its arrival observed. However, minimal subsequent entry into the TCA cycle was observed with 2-oxoglutarate at the limit of detection after acquisition with a large voxel volume and the addition of data from multiple animals^66–68^. Similarly, parahydrogen-induced hyperpolarized succinate has been utilised as a probe of primary brain cancer^69^, and it was noted that succinate was not transported sufficiently rapidly to be of utility beyond that of perfusion imaging, with no metabolic products being spectroscopically observed.

One of the defining features of later stage brain tumours is that the blood-brain barrier (BBB) has become permeabilised by dysregulated angiogenesis, mechanical disruption and inflammation, mediated by factors such as vascular endothelial growth factor (VEGF), brain angiogenesis inhibitor-1, and NF-*κβ*, amongst others^70,71^. This permeabilisation is pathological in nature, but enables the non-selective diffusion of small molecules into the brain and, hence, may increase the visibility of tumour burden to hyperpolarised imaging. In contrast to primary tumours, metastatic disease in the brain initiates through the adhesion and extravasation of tumour cells through the cerebral endothelium, with perivascular growth fed by diffusion. Continued growth leads to invasion of the brain parencheyma, which is unsustainable through diffusion alone and leads to angiogenesis. It is this angiogenesis, along with the bulk effects of tumour growth which leads to compromise of the BBB, and corresponds to a diffuse, metastatic phenotype in which the disruption of the BBB as visualised by clinical gadolinium-enhanced MR imaging corresponds to a significant tumour burden, which is associated with an exceptionally poor prognosis^72^.

Given conflicting reports in the literature about the degree of transport of hyperpolarized pyruvate across the blood-brain barrier in naive and diseased brain in animals^73–75^, we sought to investigate, and provide bounded lower limits on, the kinetics of pyruvate transport in the anaesthetised animal brain. As clinically translating hyperpolarisation methods have shown a substantially different metabolic signature from the anaesthetised animal, we wished to investigate the effect of BBB transport on hyperpolarised [1-^13^C]pyruvate experiments and determine if the probe is unambiguously metabolised in the brain parenchyma. Moreover, should that be the case, we then hypothesised that it may be possible to resolve cancer that had metastasised to the brain early, through its metabolic phenotype, prior to the point of angiogenesis, which describes a typical animal study in which the technique may be used. We were explicitly mindful of the potentially confounding effects of the large hyperpolarised signal present in the vasculature, predisposition of MR imaging techniques to partial volume effects, and high cerebrovascular perfusion.

## Results

### Rodent brain hyperpolarised [1-^13^C]pyruvate MRS

**Figure 1:**
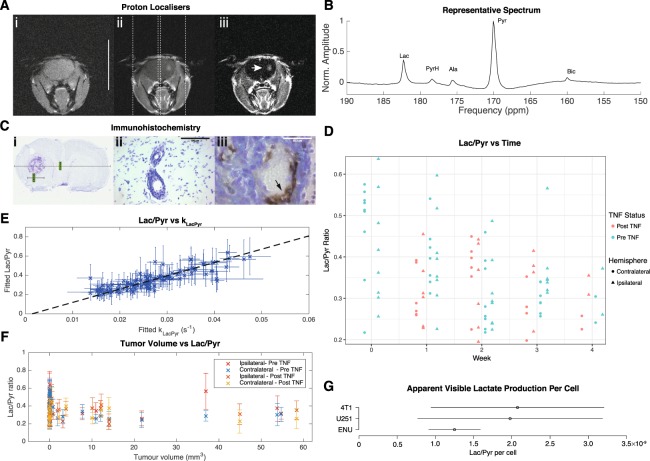
Hyperpolarised [1-^13^C]pyruvate and a model of cancer metastasis to the brain. (**A**) Three weeks after model induction via IC injection, the presence of blood-brain barrier disruption is visible via gadolinium enhanced T1 weighted MRI, shown prior to (i) and post (ii) gadolinium administration, with the difference clearly revealing the region of disease (iii). The two spectroscopic slices chosen are additionally shown; scale bar 20 mm. (**B**) Representative ipsilateral spectrum acquired from the region of disease, with lactate, pyruvate hydrate, alanine and bicarbonate visible following the infusion of hyperpolarised [1-^13^C]pyruvate. (**C**) Immunohistochemical staining of the tumour in (**A**) reveals the presence of disease near the injection site, (i) here occupying approximately a 3.6 mm^3^ area within the striatum. (ii) Cells with a metastatic phenotype can be seen growing along the perivascular niche, which express (iii) TNF receptor I along the inside of the capillary bed in the region of disease (arrow; brown). (**D**) Despite the histologically-confirmed presence of disease, we did not detect a significant difference in the lactate-to-pyruvate ratio at any time points between the affected and control hemispheres of the brain although the ratio decreased over time. TNF administration likewise did not significantly alter the lactate-to-pyruvate ratio observed. (**E**) After fitting to a pseudo-first-order kinetic model, we found that the observed lactate/pyruvate ratio correlated significantly and positively with the returned rate constant (Spearman’s *ρ* ≈ 0.9; regression line shown). (**F**) In contrast to previously published work, and our expectations, we did not observe any significant correlation of apparent lactate production with tumour volume. (**G**) In culture, however the ENU-1564 cell line injected was highly glycolytic and comparable to other reported highly glycolytic cell lines.

We initially performed slice-selective hyperpolarised [1-^13^C]pyruvate MRS weekly on female BD-IX rats following the induction of a well-characterised intracerebral model of breast carcinoma brain metastases using ENU-1564 cells over a four-week period, with visible disruption of the BBB present at approximately week three. Slice-selective spectra were obtained from both the ipsilateral and contralateral site of injection. The volume of leaking tumour was calculated from T_1_-weighted MRI pre/post gadolinium contrast (Fig. 1A).

We could resolve metabolism in the anaesthetised brain, with the detection of all peaks aside from carbon dioxide, including bicarbonate (Fig. 1B). However, the total lactate/pyruvate ratio was not significantly different between ipsilateral and contralateral hemispheres despite the presence of metastases as confirmed by histology (Fig. 1C) even at the final time-point (*p* = 0.7; Fig. 1D). As reported previously, we have found that the pseudo-first order rate constant *k*_Lac→Pyr_ was highly correlated with the lactate/pyruvate ratio (Fig. 1E)^76^. The lactate/pyruvate ratio was significantly reduced over time (*p* = 0.0037 via an anova by linear mixed effects modelling), which we believe to be consistent with an immune response following surgery, given that M1 monocytes and macrophages are highly glycolytic and produce a significant hyperpolarised lactate signal^77^. However, the subsequent decrease in lactate/pyruvate ratio over time may also show the later induction of an anti-inflammatory M2 phenotype, known to utilise fatty acid oxidation to fuel their metabolic needs^78^. Unexpectedly, no significant correlation was observed between the lactate/pyruvate ratio and tumour volume (Fig. 1F).

To assess the effect of physiologically permeabilising the blood-brain barrier, the pleiotropic cytokine TNF was injected IV two hours prior to spectroscopy at all but the “Week 0” timepoint. TNF administration has previously been shown to selectively permeabilise the blood-brain barrier in the region of metastatic disease via Gd-contrast and radio-labelled trastuzumab^79^, and has reported clinical use in limb-sparing surgery to improve chemotherapeutic uptake via direct arterial perfusion^80^. Thus, we hypothesised that the kinetics of hyperpolarised pyruvate transport and metabolism may change within the tumour-bearing hemisphere when TNF was administered. However, no significant differences were observed in pyruvate-lactate exchange, time-to-peak pyruvate or any other kinetic parameters in either ipsilateral or contralateral hemispheres following TNF administration, despite the immunohistochemical increased presence of endothelial TNF receptor 1 in the perivascular niche in the region of disease (Fig. 1C,D).

To provide a quantitative estimate for what we believe would be the lowest detectable tumour size visible to the technique, we cultured both the ENU-1564 cells injected *in vivo* and, additionally, two further cells lines; a very invasive human glioblastoma cell line reported to have a glycolytic and hypoxic profile^81–83^; and a metastatic mouse mammary carcinoma cell line, 4T1^84,85^. We found that the lactate-to-pyruvate ratio of the three cell lines considered was consistent, with no significant difference between the groups (unadjusted *p* ≈ 0.96, 0.60, 0.62 via pairwise unequal variance *t*-tests), despite the differing origins and species of the originating cell lines, with a mean value of 1.3 ± 0.6 × 10^−9^ per cell for ENU cells Fig. 1G). Under these circumstances, we would expect to be statistically powered to detect differences on the order of 3 × 10^−6^ (*α* = 0.5 using Cohen’s *d*; calculation performed in R^86–88^), which is well within the range of inter-cell line variation in total lactate efflux reported by metabolomic analysis^89^. We therefore note that these results would imply that under a set of assumptions detailed in the methods section, if perfusion differences were not limiting we would be approximately sensitive to tumours of diameter ≥1.5 mm^3^.

### Rodent brain imaging with [1-^13^C]pyruvate and ethyl-[1-^13^C]pyruvate

**Figure 2:**
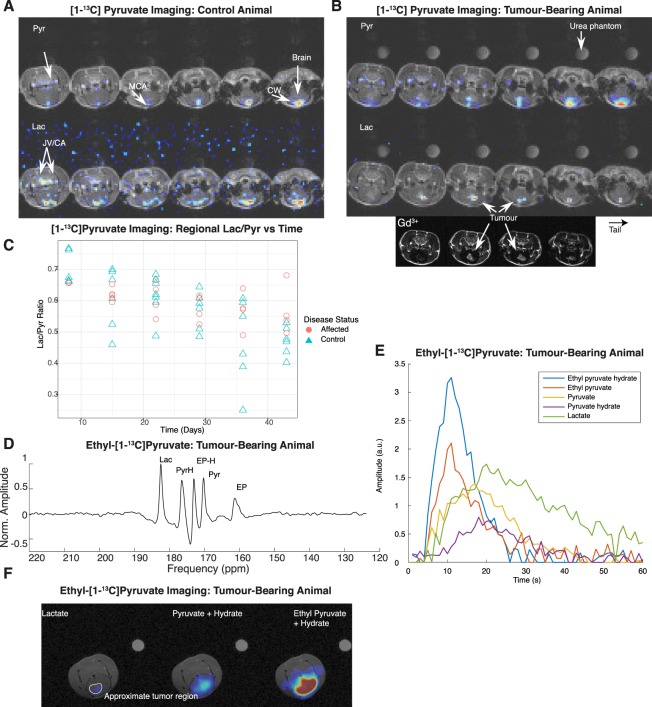
Hyperpolarised [1-^13^C]pyruvate and ethyl-[1-^13^C]pyruvate imaging in rats. (**A**) Representative summed [1-^13^C]pyruvate and [1-^13^C]lactate volumes shown concatenated through the slice axis following [1-^13^C]pyruvate infusion. Pyruvate was seen perfusing in the approximate location of the Circle of Willis (CW) and the middle cerebral artery (MCA), along with the common carotid and the jugular vein (JV/CA). The observed lactate signal is of low SNR and approximately seen in the location of the Circle of Willis. Proton images are deliberately scaled to the same resolution as the hyperpolarized scan. (**B**) As above, but for a tumour-bearing animal, shown here at day 36, together with gadolinium difference image. Lactate was observed in the tumour. The colour axis is as for A Both image stacks have a reconstructed resolution of 1 × 1 × 2 mm^3^. The regional lac/pyr ratio was not significantly different between control and affected animals, although it significantly reduced over time (*p* = 0.00795 via an anova upon linear mixed effects modelling). (**D**,**E**) Example summed spectrum and peak intensity timecourses from ethyl-[1-^13^C]pyruvate infusion, after 10 Hz exponential apodization and polynomial baseline correction. Downstream metabolites are clearly visible, with the exception of bicarbonate and CO_2_ which were not resolved. (**F**) In contrast to the predominantly vascular images of hyperpolarized [1-^13^C]pyruvate, ethyl-[1-^13^C]pyruvate freely diffuses into the brain, producing downstream pyruvate and lactate visible only in the region of disease.

To assess and determine the spatial localisation of the hyperpolarised signal received in the preceding MRS experiments, we performed longitudinal, three-dimensional, and high resolution spectral-spatial metabolic imaging on the naïve and diseased rat brain with hyperpolarised [1-^13^C]pyruvate. We observed pyruvate localising in the vasculature in the rat head, predominantly in the region of the Circle of Willis, internal and external carotid arteries, and jugular veins. Interestingly, the small amount of ^13^C-lactate present at early time points was also observed approximately in the same vessels, at the detection limit of the technique in control animals (Fig. 2A). We observed pyruvate signal in both the dorsal and ventral great vessels, indicating that the surface receive coil used in this study was not limiting. Despite the early induction of metastatic disease in a known location, it was not possible to resolve a hyperpolarized ^13^C-lactate signal until after the disease was visible by conventional gadolinium-enhanced MR, at the point of BBB breakdown. At this point, hyperpolarised ^13^C-lactate was clearly visible localised to the region of disease (Fig. 2B). However, we found that the technique was not able to distinguish between diseased and healthy tissue prior to the point of blood-brain barrier breakdown, and the average lactate/pyruvate ratio in a region of interest around the injection site drawn by a blinded operator did not show elevated lactate production (Fig. 2C).

We hypothesised therefore that transportation across the BBB was rate limiting, and precluded the detection of cancer through its metabolic phenotype prior to the point at which it would conventionally be visible. To test this hypothesis, we therefore used hyperpolarised ethyl-[1-^13^C]pyruvate, a lipophilic analogue of pyruvate that has previously been reported to cross the BBB with greater efficiency than pyruvate prior to its enzymatic conversion into [1-^13^C]pyruvate through non-specific esterases^61,90^. We hyperpolarised ethyl-[1-^13^C]pyruvate with the electronic free radical AH111501 and MultiHance (gadobenate dimeglumine) to improve solubility and glassing properties, and reached a nuclear polarisation of ~39%, approximately ~70% of that of [1-^13^C]pyruvate under similar conditions. Preliminary spectroscopic investigations revealed that we could detect ethyl pyruvate with good SNR in the brain (c.f. Fig. 2D, whose peak SNR is 56 compared with a SNR of ~80 for Fig. 1B) and with temporal dynamics that were consistent with endogenous metabolic production of lactate (Fig. 2E).

When imaged with a spiral IDEAL sequence at 2 × 2 mm in-plane resolution we found that with the same receive hardware hyperpolarised ethyl-[1-^13^C]pyruvate produced [1-^13^C]pyruvate within the brain parenchyma that was not confined only to the location of the vasculature, but approximately uniformly across the brain as a whole (Fig. 2D). Across every animal scanned, the ethyl-[1-^13^C]pyruvate derived [1-^13^C]pyruvate map was quantitatively dissimilar from that obtained via hyperpolarised [1-^13^C]pyruvate injection alone (mean Jaccard index <0.03 ± 0.02). However, as for [1-^13^C]pyruvate, lactate derived from hyperpolarised ethyl-[1-^13^C]pyruvate was only visible at the same timepoint as BBB breakdown, and was again wholly constrained to the region of disease.

### Naïve porcine brain imaging following intracarotid infusion of hyperpolarised [1-^13^C]pyruvate

**Figure 3:**
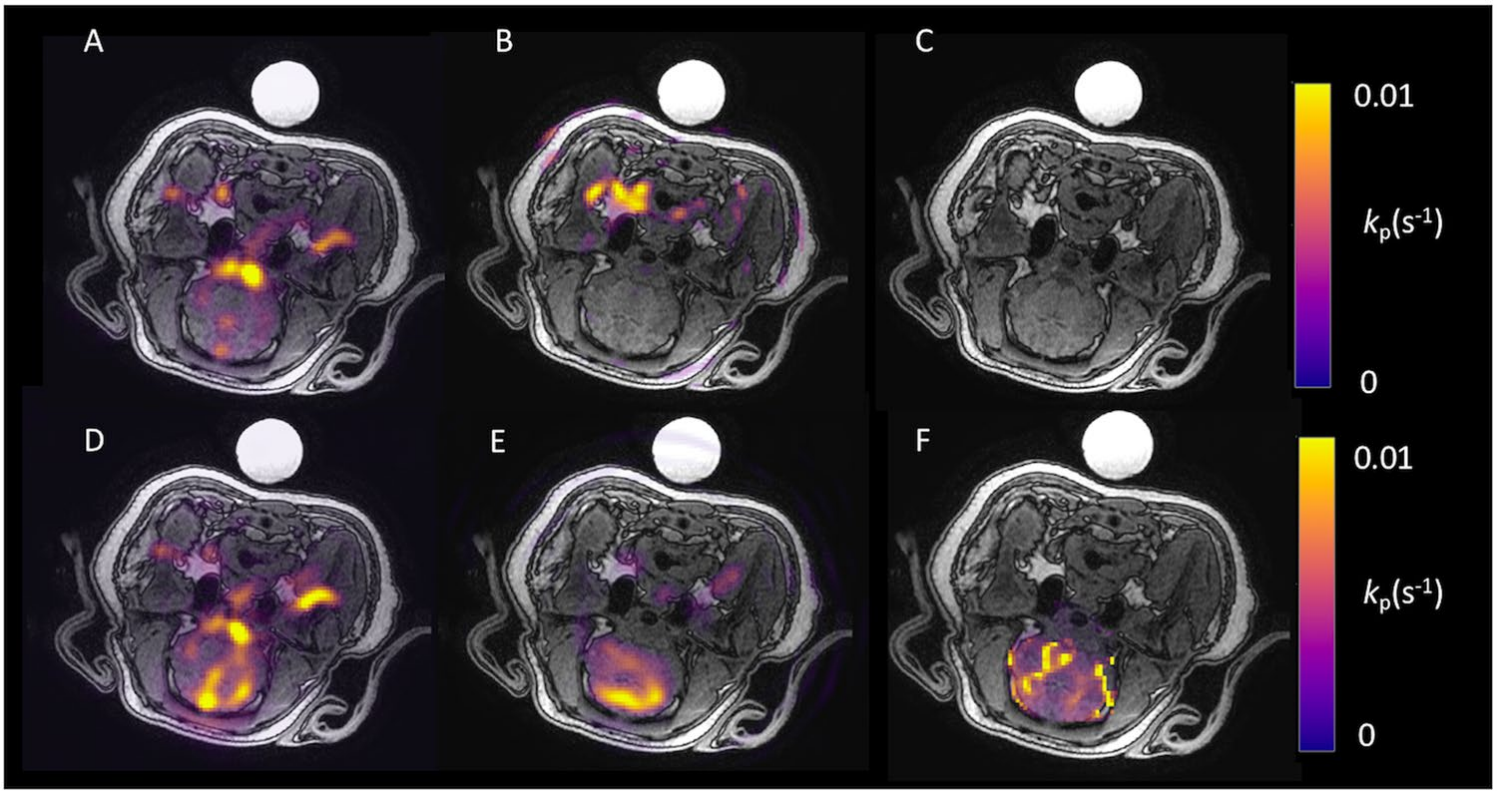
Hyperpolarised ^13^C imaging in the porcine brain. (**A**–**C**) Example summed [1-^13^C]pyruvate, summed [1-^13^C]lactate and *k*_*P*_ maps of the porcine brain post saline infusion, respectively. Here no detectable lactate signal is observed post bolus, with [1-^13^C]pyruvate appearing in the major vessels of the brain. (**D**–**F**) Representative [1-^13^C]pyruvate, [1-^13^C]lactate, and *k*_*P*_ of the porcine brain post mannitol infusion, respectively. Here lactate exchange is observed globally, post infusion, with a similar distribution of [1-^13^C]pyruvate signal in the major vessels as seen in (**A**). The object seen in the posterior of the pig is the [1-^13^C bicarbonate] sphere used for transmit gain and centre frequency calibration.

To further investigate the potential utility of cerebral pre-clinical imaging, we injected 20 ml of hyperpolarised [1-^13^C]pyruvate prepared in the Spinlab polariser currently used in clinical studies and via an angiography catheter directly into the internal carotid artery of anaesthetised healthy swine prior to metabolic imaging at 6 × 6 × 55 mm^3^. We observed cerebral perfusion through the Circle of Willis, posterior, anterior and superior cerebellar arteries, middle cerebral artery, and into the basilar arteries. A small amount of concomitant lactate production was observed, but not specifically localised to the brain parenchyma. Bounded kinetic analysis revealed no exchange of pyruvate to lactate (c.f. Fig. 3A–C) within the brain, i.e. *k*_*p*_ = *k*_Pyr→Lac_ = 0.

### Porcine brain imaging following mannitol infusion

**Figure 4:**
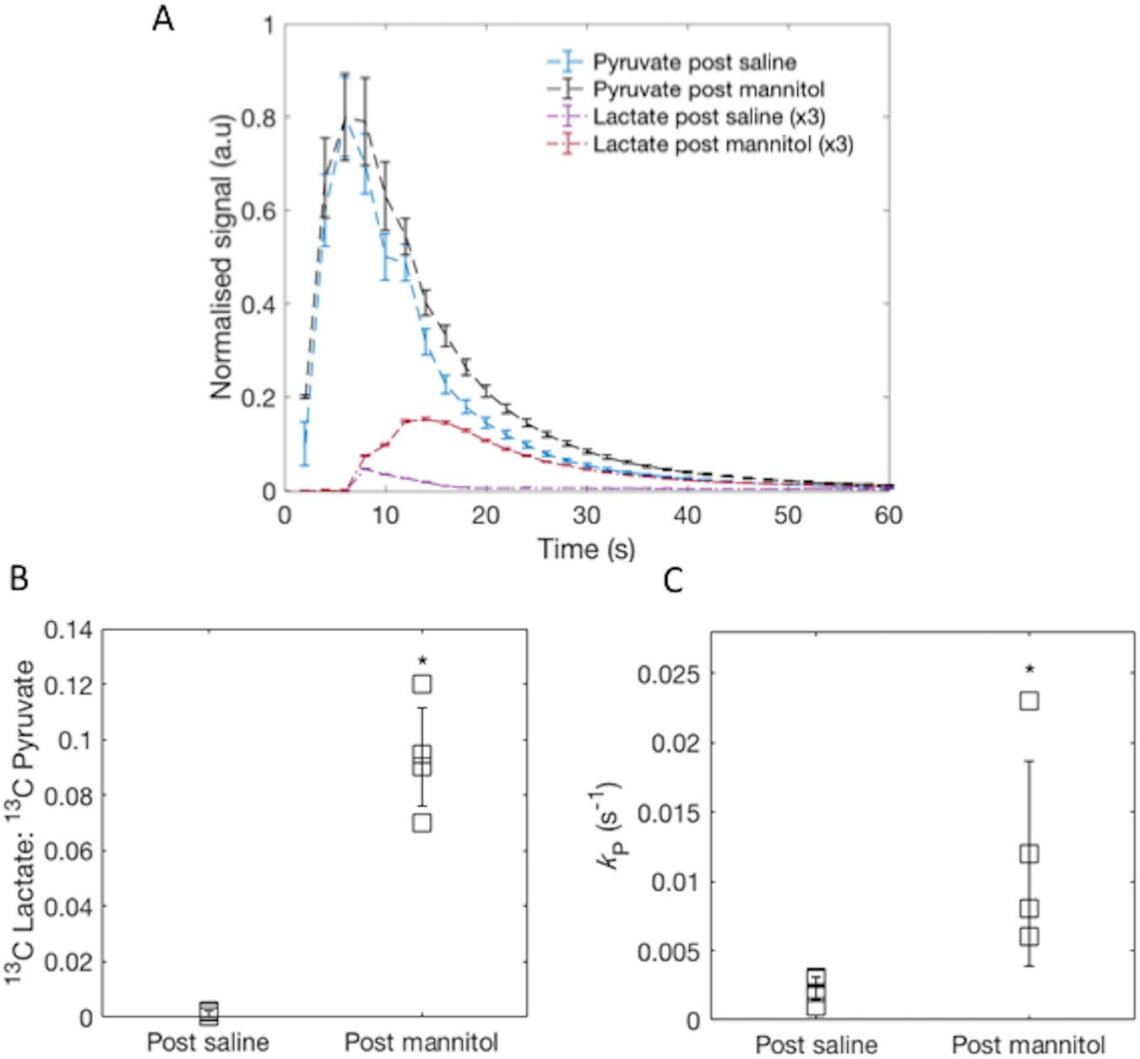
Kinetic analysis of the porcine brain. (**A**) Average [1-^13^C]pyruvate and [1-^13^C]lactate time course data from regions of interest placed in porcine brains reveals inflow and decay of the [1-^13^C]pyruvate signal post saline and mannitol. Lactate exchange is observed post mannitol infusion. Lactate normalised to peak [1-^13^C]pyruvate signal in both cases, displayed (x3) for ease of viewing. (**B**) Average [1-^13^C]lactate to [1-^13^C]pyruvate ratio for all porcine experiments An increase in the exchange of [1-^13^C]lactate from the increased permeability of the blood-brain barrier is observed (*p < 0.05, effect size = 31, mean ± SD). (**C**) Increase in *k*_*P*_ after disruption of the blood- brain barrier (*p < 0.05, effect size = 4.5, mean ± SD).

To osmotically disrupt the blood-brain barrier (BBB), anaesthetised swine were infused with mannitol via the previously placed intracerebral catheter, and a further hyperpolarised [1-^13^C]pyruvate injection undertaken immediately. In stark contrast to experiments conducted in the presence of an intact BBB, we observed significant exchange of [1-^13^C]pyruvate to [1-^13^C]lactate within the cerebrum as a whole, and in particular the proximal and rostral portions of the parietal lobe. Furthermore, *k*_*p*_ was detected in the brain, see Figs 3D–F and 4A. We found that mannitol disruption did not impede hyperpolarised experiments, which occur on a timescale of approximately three minutes.

Within the mannitol-permeabilised brain the [1-^13^C]lactate to [1-^13^C]pyruvate ratio was significantly higher than that in the naïve brain (0.09 ± 0.02 vs 0.001 ± 0.001, respectively, p < 0.05, Cohen’s *d* effect size = 31). *k*_*p*_ was also increased (0.012 ± 0.07 vs 0.002 ± 0.001, p < 0.05, effect size = 4.5), c.f. Fig. 4B,C. No [1-^13^C bicarbonate] was observed in the porcine brain in either case.

## Discussion

There are a large number of neurological pathologies in which metabolism is dysregulated where hyperpolarised imaging may therefore be of direct utility. We initially wished to use longitudinal hyperpolarised [1-^13^C]pyruvate imaging to detect a rodent model of cancer metastasis to the brain by its metabolic phenotype before its direct detection via gadolinium enhanced MRI mediated by disruption of the BBB. We found that this was not possible by either spectroscopy or imaging: increased [1-^13^C]lactate signal was only detectable after the point of BBB disruption as assessed by direct proton imaging. We note that this does not preclude preclinical utility, e.g. through the use of the technique to monitor the response of disease to therapy, but we wished to provide a physiological interpretation for the low SNR of these imaging techniques compared to other organs, especially the heart, for which high-resolution temporal imaging can be readily obtained^12,17,20^. As a consequence, we therefore investigated the effect of permeabilising the blood-brain barrier physiologically in the region of disease, in an attempt to increase pyruvate transport and hence apparent lactate production. We found that TNF did not significantly alter transport kinetics despite the histological presence of the receptor, and thus did not pursue its use further.

Our imaging experiments show clearly and reproducibly the appearance of [1-^13^C]pyruvate in the great vessels on both sides of the rat head and neck, illustrating that the receive profile of the RF coil array would not be limiting for the detection of metabolites within the brain parenchyma. In naïve rats, we apparently observed lactate production within the great vessels also, which may reflect either the glycolytic nature of red blood cells, or alternatively the efflux of hyperpolarised [1-^13^C]lactate from other organs, such as the liver^91^. We note that while we detected bicarbonate spectroscopically, we were not able to image its production. Additionally, the apparent lactate-to-pyruvate ratio obtained from cell culture of the tumour cells used *in vivo* indicated that these were indeed highly glycolytic, and comparable to other lines that are reported in the literature as being highly glycolytic obtained from a variety of species. Under a reasonable set of assumptions we analytically estimated that the SNR reduction compared to that observed in other tissues cannot necessarily be explained on physical grounds alone, as we would predict that a large glycolytic flux would be observable during the perivascular growth phase of the metastatic colony. This was not experimentally found to be the case.

The use of ethyl-[1-^13^C]pyruvate as a lipophilic analogue of pyruvate has been previously proposed^61^. In this work we have further improved the limiting polarisation reached with that molecule through the use of a more lipophilic radical and gadolinium chelate, and additionally present the first spiral IDEAL imaging of ethyl-[1-^13^C]pyruvate in the diseased brain. We clearly and unambiguously observe [1-^13^C]pyruvate production from ethyl-[1-^13^C]pyruvate in the entirety of the brain parenchyma with the same receive hardware as used previously, and additionally observe lactate production in the region of metastases at the three-week timepoint. However, the presence of the additional spectral resonances serves to make the use of spectral-spatial imaging sequences, such as that used for high resolution [1-^13^C]pyruvate imaging, challenging, owing to the reciprocal relationship between excitation bandwidth and the duration of the pulse. Instead, the spiral IDEAL approach used here has a comparatively coarse spatial resolution, but higher effective spectral resolution, and is able to spectrally, if not spatially, resolve the endogenous production of [1-^13^C]lactate in the naïve brain downstream from ethyl-[1-^13^C]pyruvate. We believe that this discrepancy arises due to the multiple metabolic steps that lactate production has to undergo, leading to a signal at the detection limit of the technique with our hardware. We note that this apparently contradicts the earlier result of Hurd *et al*.^61^, who apparently observe whole-brain lactate production throughout the whole brain. In comparison to the work of Hurd *et al*., this work was performed at higher field (7 T vs 3 T), resulting in a reduction in the available ${{\rm{T}}}_{2}^{\ast }$ for imaging and additionally a reduction in T_1_, similarly to pyruvate^92^. Ignoring differences in receive chain noise performance, our use of a 72 mm volume transmit coil provides greater transmit homogeneity, but the smaller receive elements used may not be equally sensitive to the dorsal side of the brain, which may be further expected to reduce apparent SNR from that region. We therefore believe that endogenous lactate production is slightly below the noise floor of this experiment, with the increased rate of glycolysis within the tumour being detectable.

We note that pyruvate is transported across the plasma membrane via the monocarboxylate family of proton-mediated transporters. There is substantial evidence to show that anaesthesia reduces both neuronal metabolism directly, and the rate of pyruvate transport through the brain. Both are altered under different conditions such as anaesthetic dose, type, and duration^93,94^.

On the basis of the above experiments, we hypothesised that transportation through the BBB provided by monocarboxylate transporters and subsequent metabolism may be limiting for neurological studies on anaesthetised rodents with hyperpolarised [1-^13^C]pyruvate. We note that the apparent reduction in [1-^13^C]pyruvate transport across the blood-brain barrier is consistent with previous work quantifying ^14^C-pyruvate transport in the pentobarbital anaesthetised rat brain, in which it was shown to be approximately 100-fold slower than in the rodent heart under similar conditions (Michaelis-Menten *V*_max_ = 0.18 *μ*mol min^−1^ g^−1^ in the brain vs *V*_max_ ≈ 10 *μ*mol min^−1^ g^−1^ in the heart)^95,96^.

Owing to its reported role as a neuroprotective agent, the cerebral uptake and metabolism of exogenous pyruvate has been substantially investigated, and it is regarded as an excellent substrate for cerebral energy metabolism that is predominantly metabolised by neurones^97^. However, both the rate of transport and the rate of utilisation of these substrates are reduced under anaesthesia. As differing anaesthetic regimens that alter EEG activity cause a linear and concomitant drop in neuronal metabolism^98^, the differing regimes used here – propofol and isoflurane – are not expected to substantially alter neuronal metabolism owing to their minimal effects on EEG activity^99^, and are consistent with “light” anaesthesia as reported previously^95,100^, which quantitatively does not explain this discrepancy alone.

However, we note that previous work has reported that the lactate to pyruvate ratio is decreased under isoflurane anaesthesia of increasing depth in the brain^100^, heart^101^, and kidney^102^. Additionally, the lactate to pyruvate ratio is altered profoundly following the administration of different anaesthetic agents, with a reduction in the derived metabolic rate constant with increasing depth of anaesthesia from alpha-chloralose to pentobarbital^103^. A further confounding factor is that as well as altering neuronal metabolism, it is known from radiolabelled tracer experiments that anaesthetic regimens alter the transport of pyruvate and other metabolites into the brain, separately from altering their subsequent metabolism^93^. Indeed, neuronal pyruvate uptake and oxidation rates are altered physiologically through development and ageing amongst other processes^95^, and pathologically upregulated following traumatic brain injury as mediated via an increase in MCT-2 expression^104^ as well as in cancer^7^.

We therefore wished to see whether our results were species-specific and confined to rodents, or if it persisted in larger animals scanned at clinical field strengths with comparable, but distinct anaesthetic agents. Here we have presented the first results of hyperpolarised [1-^13^C]pyruvate imaging in the anaesthetised porcine brain. Imaging revealed no discernible exchange of [1-^13^C]pyruvate to [1-^13^C]lactate in the naïve brain, mirroring the rodent case. We likewise observed the presence of [1-^13^C]pyruvate in the great vessels, but not in the brain parenchyma itself. Lactate was confined to extra-vascular spaces, and not visible in the middle cerebral artery, in contrast to the rat brain. However, by introducing mannitol under general anaesthesia to globally osmotically disrupt the blood-brain barrier we observed far greater pyruvate transport into the brain, and the clear presence of lactate within almost the entirety of the brain, with a clear kinetic shift in the resulting metabolic time-course, as well as a significant 90-fold increase in whole-brain lactate/pyruvate compared to saline injection. While the osmotic disruption of the BBB is not physiological, it is reversible^105^ and mannitol incorporation into neuronal tissue itself is comparatively slow compared to the lifetime of the hyperpolarised experiment^106^.

This procedure renders feasible the use of pigs for neurological hyperpolarised studies, undertaken using a clinical scanner and sequences, allowing for the translation of sequence methodology to future human studies. Large animal imaging on a clinical system provides a powerful tool to probe disease with similar physiological parameters, such as blood pressure, heart rate, and cerebrovascular perfusion. However, as shown here, it may be the case that the use of additional permeabilising agents (such as ultrasound or mannitol) may be required if hyperpolarised [1-^13^C]pyruvate is to adequately probe neurovascular perfusion in a pre-clinical environment. Future work will investigate more thoroughly the role of anaesthetic agents on cerebral metabolism as quantified through DNP, if possible including similar studies on awake animals adjusted previously to fMRI protocols. As the use of large animals provides the opportunity for serial imaging with multiple tracers, where rodent models may not tolerate extended protocols, the methods developed may provide a unique opportunity to understand longitudinal alterations in cerebral metabolism (as quantified through hyperpolarised [1-^13^C]pyruvate and [2-^13^C]pyruvate, amongst other molecules), perfusion ([^13^C]-Urea), and cellular necrosis ([1,4-^13^C]fumarate) in highly relevant disease models with clinically translatable hardware.

We believe that our results can only be consistent with the explanation that BBB transportation limits hyperpolarised [1-^13^C]pyruvate metabolism in anaesthetised animals. While this result apparently contradicts previously published rodent imaging work observing cerebral hyperpolarised metabolism, we note that the rat imaging sequence used here is able to resolve vascular structures in three dimensions, and that previous work using [1-^13^C]pyruvate has imaged models of primary brain cancer at comparatively modest spatial resolution, e.g. 5 × 5 × 10 mm^3^ ^63^, which would show a projection over much of the circle of Willis in the rat brain. Additionally, under these conditions, the point spread function of spectral imaging sequences is large, and Gibbs ringing and partial volume effects may not be neglected. In particular, the presence of a large vascular signal in the middle cerebral artery combined with axial imaging orientations and the sinc-shaped artefact arising from low matrix size chemical shift imaging sequences may lead to a substantial degree of spatial confounding, although we cannot discount the hypothesis that variable responses to anaesthesia may drive differing lactate kinetics.

While it may appear that the apparent lack of adequate transport across the blood-brain barrier would limit the future translational applicability of the technique, we would like to note that several recently published human studies have performed hyperpolarised [1-^13^C]pyruvate imaging in the humans brain, and resolve bicarbonate production *in vivo*^46,107^. We hypothesise, therefore, that this study raises the question of whether or not anaesthesia, or species-specific differences, are responsible for the apparent discrepancy between human and pre-clinical results. We note that the studies as undertaken have used two distinct anaesthetic agents, isoflurane/nitrous oxide and propofol/fentanyl, and observed similar limitations in both porcine and rat brain. As it has previously been noted that the depth of isoflurane anaesthesia has a depressing effect on hyperpolarised metabolite-to-substrate values in the brain and the heart in a dose-dependent fashion^100,101^, our work suggests that in preclincial studies the rate of uptake across the intact BBB is slow compared to the hyperpolarised experiment, even at the comparatively low isoflurane dose of 2% and under 0.4 mg/kg/h propofol anaesthesia. While we cannot assess directly the degree of neuronal metabolism independent of transport kinetics, the fact that mannitol permeabilisation enables the resolution of the hyperpolarised [1-^13^C]lactate signal within the brain would indicate that the hardware and imaging sequences we possess would enable the visualisation of neuronal metabolism were it not for the blood brain barrier.

We propose, therefore, that hyperpolarised [1-^13^C]pyruvate imaging studies could be of greater utility in diseases in which the blood brain barrier is expected to be focally disrupted, such as multiple sclerosis in which blood-brain barrier disruption is an early sign^108^. Additionally, we note that there exist ethical considerations that would favour the use of the technique in situations where the blood-brain barrier is already expected to be pathologically, and not physiologically, permeabilised. We urge preclinical researchers to consider the impact of pyruvate transport across the brain, quantitatively consider partial volume effects from nearby vasculature, and use appropriate anaesthetic agents. Further work will therefore aim to explore cerebral metabolism in disease with hyperpolarised MR, and additionally consider the utility of other endogenous compounds with potentially distinct transport kinetics, such as hyperpolarised [1-^13^C]lactate itself.

## Methods

### Rodent MRS and MRI

#### Animal model

Rodent experiments were carried out under appropriate personal, project and institutional licences granted under the UK Animals (Scientific Procedures) Act 1986, following ethical review both locally by the University of Oxford Clinical Medicine Ethical Review Committee and nationally as part of the project license application. All experiments were conducted in accordance with the University of Oxford Policy on the Use of Animals in Scientific Research. For model induction, female BD-IX rats (*n* = 8) were anaesthetised with isoflurane (4% induction for approximately 1.5 min, 2% maintenance) and tumours aseptically induced by stereotactic intracerebral (IC) injection of approximately 5000 ENU-1564 cells resuspended in PBS at 1 mm anterior, 2.9 mm left of Bregma, at 1.5 mm and 2.5 mm deep. A control contralateral injection of an equivalent volume of sterile PBS was also performed. The ENU-1564 cell line was chosen as a rat model of brain metastasis from a mammary carcinoma^109–112^.

Rats were housed in accordance with institutional guidelines, allowed food and water *ad libitum* and scanned only in the afternoon. During IC surgery, rats were administered with subcutaneous bupivacaine hydrochloride and adrenaline (Marcaine) for postoperative analgesia and monitored daily postoperatively for neurological symptoms, which were chosen to mark the humane end-point. At the last time-point animals were sacrificed via anaesthetic overdose (250 mg pentobarbital under isoflurane anaesthesia) followed by cardiac perfusion with saline and periodate lysine paraformaldehyde fixative with 0.025% w/v glutaraldehyde (PLP_*light*_) as described previously^109^.

#### Immunohistochemistry

Brains were removed, fixed for a further 4–6 hours in (PLP_*light*_), left to cryoprotect in 30% w/v sucrose, frozen, and then sectioned. The expression of TNF type I receptors was confirmed by immunohistochemistry. Briefly, frozen 10 coronal sections were cut through the brain close to the injection site; slides were dehydrated in ethanol and endogenous peroxidases blocked by immersion in 0.3% H_2_O_2_ in methanol with general blocking provided by goat serum. Sections were stained with rabbit anti-TNF-I as primary antibodies (New England Biolabs, UK), using a standard protocol, and then counterstained with Cresyl Violet ((9-dimethylamino-10-methyl-benzo[a]phenoxazin-5-ylidene)ammonium chloride; a nuclei stain). Primary antibodies were applied and incubated overnight. A biotinylated anti-fluorescein antibody was then applied to the sections, and incubated overnight before detection using a commercial “ABC” amplification system (Vector Laboratories, UK). The application of diaminobenamidine (Vector Laboratories, UK) in the presence of the catalyst imidazole (0.01 M) evolves a dark brown colour in the presence of the primary antibody.

#### Rodent ^13^C MRS and ^1^H MRI

Rats were scanned weekly starting one day following surgery until the appearance of any neurological symptoms, for a total of 102 observations across five weeks. Animals were anaesthetised in isoflurane (3% induction for approximately 1.5 min, 2% maintenance) and placed supine onto a home-built 30 mm ^13^C loop coil in a home-built homeothermic small animal MR cradle and scanned on a Varian/Agilent 7 T preclinical MRI system. Although we note that it may have been advantageous to titrate anaesthetic dose automatically to a target respiration rate, we found that the constant-flow anaesthetic gas system used effectively resulted in a mean respiratory rate of approximately 60 bpm with a standard deviation of 10 bpm. The centre of the coil was localised to the surgical site. T_1_-weighted gradient echo axial proton images were acquired (TR 120; 50 bandwidth; TE 4.8 ms; FA 60°; 40 × 40 mm^2^ FOV; 192 × 192 matrix) and a multi-echo based 3D automated shimming algorithm used to ensure *B*_0_ homogeneity over the brain. [1-^13^C]Pyruvate was mixed with OX063 radical and hyperpolarised using a prototype hyperpolariser at 1.4 K, and 3.35 T/94 GHz as described previously^113^. After dissolution, 1 ml of hyperpolarised pyruvate (approximately 80 mol total dose) was injected via a previously placed tail vein cannula. Sagittal slice-selective spectra were subsequently obtained interleaved from both the contralateral and ipsilateral hemispheres of the brain with a 10 mm slice thickness, and a 1 mm gap excluding both the corpus callosum and middle cerebral artery. Spectra were acquired as described previously^114^; briefly, with a nominal 15° 350 μs sinc excitation, 1 s TR, 1024 complex points and a 8012 Hz spectral bandwidth. Spectral quantification was undertaken with the AMARES algorithm and error propagation as described previously^115^.

After the hyperpolarised injection, gadolinium was administered (Omniscan/Gadodiamide; 1.35 mmol kg^−1^) and an additional set of gradient-echo proton images acquired to assess tumour burden. Rat TNF was administered *i*.*v*. (60 μg kg^−1^; New England BioLabs, UK) and each animal allowed to recover for two hours prior to subsequent spectroscopy.

#### Hyperpolarised [1-^13^C]pyruvate imaging

To accurately assess the spatial distribution of hyperpolarised pyruvate, a separate population of rats were subject to the same surgical induction described above (*n* = 4) or injection with control vehicle (*n* = 4), anaesthetised and scanned weekly after IC injection. The proton protocol was the same as above. For hyperpolarised imaging, in order to resolve a greater spatial extent of [1-^13^C]pyruvate transport, a dual-tuned proton/carbon 72 mm volume transmit birdcage coil was used with a two-channel 40 mm surface receive array with an integrated preamp. A flyback 3D spectral-spatial EPI sequence (37.5 mm slab excite; 64 × 64 mm^2^ FOV; volume ^13^C transmit/surface receive; TR 150 ms; TE 12.18 ms) was designed and reconstructed as described previously^12,115^ at 2 × 2 × 4 mm^3^. 31 mg of [1-^13^C]pyruvate with OX063 radical was polarized in a prototype hyperpolarizer for 45 minutes prior to dissolution as described previously^116^, and injection of 2 ml 80 mmol pyruvate performed over 20 s via a tail vein cannula. Hyperpolarized pyruvate/bicarbonate/lactate volumes were acquired interleaved (TR = 1.8 s/volume; total FA = 17°, 61°, 61°). After reconstruction, a ~2 mm^3^ region of interest was drawn around the injection site by an operator blinded to the injection compound.

#### Cell experiments

As an *in vitro* control to directly measure the apparent flux through lactate dehydrogenase, the ENU 1564 cell line used in the *in vivo* experiments along with two other types of cancer cells (U251, a highly glycolytic glioblastoma cell line; and 4T1 cells, derived from circulating tumour cells of a mouse mammary carcinoma) were grown in culture. Dulbecco’s Modified Eagle Medium with 10% (v/v) FBS and 1% (w/v) glutamine was used as culture medium with 1% penicillin and streptomycin solution (w/v). After thawing, cells were grown in a T-25 flask and incubated at 37 °C until 80% confluent, before being split into a T-175 flask with 0.05% (w/v) trypsin. Cells were pelleted by centrifugation at 700 × *g*, and re-suspended in 1 mL complete medium at 37 °C in a warmed 1 cm diameter NMR tube.

The warmed NMR tube was then transported in a bottle of warm water to a heat bath next to a 500 MHz Bruker Avance spectrometer, positioned adjacent to a ‘Hypersense’ (commercially available) DNP hyperpolariser. After shimming on a tube containing an equal volume of water, the cell suspensions were placed in the spectrometer. The dissolution was performed and the evolved liquid diluted threefold into a buffer containing 300 μM K^+^-EDTA, before 1 mL was injected directly into the cell suspension. Simple pulse-acquire spectroscopy (hard pulse, 15° flip, TR 1 s) was then used to quantify the rate of lactate production following the injection of hyperpolarised [1-^13^C]pyruvate. Three repeats were made for each measurement obtained from three different culture flasks. The RF coil used was a dual-tuned proton/carbon birdcage probe, of diameter ~10 mm and length 14 mm.

After the dissolution, the number of live cells present in the NMR tube was estimated by sampling 12.5 μL volumes from the total volume, and mixing with the diazo dye Trypan Blue to differentially stain live and dead cells prior to automatic counting by an Invitrogen ‘Countess’ hæmocytometer. Approximately 1 × 10^6^ to 1 × 10^7^ cells were used per experiment for measurable lactate production. Spectra were temporally summed prior to quantification with AMARES as described previously^115^. The limit of detection for metabolic signal was approximately 1 × 10^6^ cells resuspended in 2 ml total volume.

To provide an approximate estimate of the expected SNR per unit cell, we wished to analytically estimate the difference in reception sensitivity between the *in vivo* and *in vitro* settings, and thus get a very approximate idea of the relative contribution of perfusion effects. For both the *in vitro* and *in vivo* case, the MR coils used are small, and are coil-noise dominated and not sample-noise or preamplifier shot-noise dominated. It is well-known that the SNR in single-coil magnetic resonance experiments scales as

$${\rm{SNR}}=\frac{\sqrt{2}\omega {M}_{0}{B}_{1}}{\sqrt{4{k}_{B}RT}}$$

where *k*_*B*_ is Boltzmann’s constant, *R* the resistance of the detection coil, *T* the temperature of the sample, *M*_0_ longitudinal magnetisation and *B*_1_ is the receive field, assumed here through the principle of reciprocity to be equal to that of the transmit field^117^. For the high-field birdcage coil used, it is known that *B*_1_ can be expressed as^118^

$${B}_{1{\rm{m}}{\rm{e}}{\rm{a}}{\rm{n}},{\rm{b}}{\rm{i}}{\rm{r}}{\rm{d}}{\rm{c}}{\rm{a}}{\rm{g}}{\rm{e}}}=\frac{2{\mu }_{0}I\zeta }{\pi d}\frac{l}{\sqrt{{l}^{2}+{d}^{2}}}(1+\frac{{d}^{2}}{{l}^{2}+{d}^{2}})$$

where *l* is the length of the resonator, *d* its diameter, *I* the current flowing in the coil, and $\zeta$ a function of the rung spacing of the coil, which we here estimate as being approximately 0.77 based on numerical integration procedures detailed elsewhere^119^. For the loop coil on axis, it is also well known that in the quasi-static region Biot-Savart provides an expression for the field as

$${B}_{1{\rm{p}}{\rm{o}}{\rm{i}}{\rm{n}}{\rm{t}},{\rm{l}}{\rm{o}}{\rm{o}}{\rm{p}}}=\frac{{\mu }_{0}}{2}\frac{{r}^{2}I}{{({z}^{2}+{r}^{2})}^{3/2}}$$

where *r* is the coil’s radius, and *z* the distance from the axis. Under conditions whereby a defined flip angle is delivered, the current that flows is limited by the amplifier power *W*, i.e. $I=\sqrt{W/R}$, which is typically limited by either the excitation bandwidth desired or the available *W*^120^. Considering geometry factors alone we calculate that the 30 mm loop coil is approximately a factor of 3.37 times worse in SNR for a small volume axially 10 mm away from the loop, i.e. the approximate distance of the tumour from the coil. Including the effects due to the difference in *B*_0_, we therefore end up with a very approximate statement that as *M*_0_ is independent of *B*_0_ in the hyperpolarised case, we should expect SNR to be ~17% on the 7 T experiments compared to those at 11.7 T. This, combined with an experimentally derived limit of detection of ~1 × 10^6^ cells at 11.7 T in the 14 mm sensitive region of the coil provides an estimate of a minimum “detectable” tumour size of 1.48 mm^3^, assuming a cell density of 2.8 × 10^6^/mm^3^ as experimentally derived via semi-automated cell counting on histology slides.

#### Hyperpolarised ethyl-[1-^13^C]pyruvate imaging

Owing to the increased lipophilicity of ethyl-pyruvate compared to pyruvate, we investigated the use of the more lipophilic AH111501 EPA radical in contrast to OX063. Ethyl-[1-^13^C]pyruvate was obtained from Sigma Aldrich Ltd. (Gillingham, UK), and was prepared as neat acid doped with 15 mM AH111501 “EPA” trityl radical. To 30 μl of this solution was added 5 μl of 10 mM Dotarem (Guerbet Laboratories Ltd) gadolinium chelate solution (2% volume-to-volume in 100% ethanol). The resulting solution was hyperpolarised in a prototype DNP hyperpolariser at 93.965 GHz and 100 mW for approximately 150 minutes. Dissolution was performed at a pressure of 10 bar and a temperature of ~170 °C in 4.5 ml of 0.03 mM tris(hydroxyl-methyl) aminomethane (TRIS) with 0.25 mM dipotassium ethylenediaminetetraacetate (EDTA) buffer solution resulting in a pH ~ 7 solution of approximately 54 mM ethyl-[1-^13^C]pyruvate. 2 mL of this solution was then injected over 20 seconds via the tail vein. The time delay between dissolution and injection into the animal ranged from 20 to 25 seconds. We found that the limiting solid state polarization reached by ethyl-[1-^13^C]pyruvate was approximately 73% of that of pyruvate, with a comparable liquid-state *T*_1_. A spiral multiecho sequence was designed with echo times to maximise the total effective number of signal averages over all metabolites (30 mm sinc excitation; 80 × 80 mm^2^ FOV; 0.5 s TR; 8 echoes; ${\rm{TE}}=1.05,1.59,2.13,2.67,3.21,3.75,4.29,4.83$, with a separate spectral FID 64 ms, FA/shot = 15°). The same volume transmit/surface receive coil hardware was used as for [1-^13^C]pyruvate imaging. Data were reconstructed by a pre-measured gradient impulse response function^121^ followed by NUFFT^122^ for a reconstructed resolution of $3.125\times 3.125\,{{\rm{mm}}}^{2}$. Multicoil data was recombined via the method of McKenzie^123^. The ideal reconstruction was performed via explicit calculation of the Fourier matrix.

### Porcine MRI

All porcine imaging was undertaken in accordance with the Danish Animal Welfare Act 2013 following an explicit national ethical review process undertaken by the Danish Animal Experiments Inspectorate. Four healthy adult pigs (Female Danish domestic pigs, average weight 30 kg) were anaesthetised with a continuous infusion of propofol (12 mg initial dose, 0.4 mg/kg/h thereafter) and a 5-French angiography catheter was introduced into the common carotid artery under X-ray guidance. Imaging was undertaken on a GE 3 T HDX clinical scanner (GE Healthcare, Waukesah, WI, USA) with subjects placed supine. Proton imaging was performed with an 8 channel array coil (GE, Healthcare, USA) with a 3D T_1_ weighted gradient echo sequence with, TR = 4.9 ms, TE = 2.3 ms, FOV = 240 mm, FA = 14°, resolution = 1 × 1 × 1 mm^3^). ^13^C imaging was undertaken with a Helmholtz loop pair (Pulse Teq, Chobham, UK) placed anterior and posterior to the skull. Transmit gain and centre frequency were calibrated using a spherically symmetric 2 M ^13^C -bicarbonate phantom placed in the coils’ field of view, using a Bloch-Sigert method^124^.

The hyperpolarisation protocol was as described previously^125^, scaled proportionally for body mass: 0.64 g of [1-^13^C]-pyruvic acid (Sigma-Aldrich) was mixed with EPA (15 mM, Syncom) and was hyperpolarised in a GE SPINLab for approximately three hours. Two injections were administered prior to hyperpolarised imaging: a saline control infusion followed one hour later by mannitol. The control saline injection was undertaken as a 300 ml bolus at 8 mL s^−1^ followed by a 40 ml chaser of sterile saline, and then one hour subsequently 300 ml of 1.4 M mannitol (Sigma-Aldrich) was infused at 8 mL s^−1^ via the catheter to induce blood-brain barrier permeabilisation, followed by a subsequent 40 ml saline flush. Sixty seconds after the end of either flush, 20 ml of 250 mM [1-^13^C]pyruvate (pH ≈ 7.5) was injected directly into the previously placed catheter (5 ml s^−1^). Hyperpolarised ^13^C imaging was performed using an IDEAL spiral CSI sequence with interleaved spectrum acquisition as described above and previously^126^ (axial 2D sices, TE = 1.1 ms, ΔTE = 1.1 ms, TR = 250 ms, 7 echoes and one FID, slice thickness = 55 mm, in-plane resolution = 6 mm^2^ time resolution = 2 s, total imaging time = 48 s). Imaging began at the end of the hyperpolarized injection.

### Quantitative post processing

Dynamic images and spectra were summed in the complex domain, and ratiometic maps of lactate:pyruvate, were produced. Images were fitted in the frequency domain to produce *k*_*P*_ maps^127^. A region of interest was drawn in the brain of each case, and used for average measurements of [1-^13^C]lactate: [1-^13^C]pyruvate and *k*_*P*_.

### Statistical analyses

Rodent statistical analyses were performed in R, with the linear mixed effects package lme4 used for timecourse analysis^128^, with Anova analysis provided through the car package^129^, and other procedures provided by the core features of the language. Porcine statistical analysis was undertaken in Matlab using the Statistics and Machine Learning toolbox, to assess for differences between pre–and post-mannitol kinetic results. In all cases, the conventional significance level of *p* < 0.05 was used, all tests were two-sided, and Cohen’s *d* effect sizes are reported where appropriate. Although we believe that linear mixed effects modelling is the most appropriate for the longitudinal data provided by the rodent study with each rodent being considered a random effect, the lack of significance reported is also found via conventional linear modelling/ANOVA or Kruskal-Wallis non-parametric ordinal logistic regression.
